# Capillary hemangioma of cauda equina: a case report

**DOI:** 10.1186/1757-1626-2-80

**Published:** 2009-01-22

**Authors:** Seyed M Miri, Zohreh Habibi, Mohammad Hashemi, Ali T Meybodi, Seyed Ali F Tabatabai

**Affiliations:** 1 Department of Neurosurgery, Imam Khomeini Hospital, Tehran University of Medical Sciences, Tehran, Iran

## Abstract

**Background:**

Capillary hemangiomas of spinal nerve root, mostly affecting the cauda equina, are extremely rare.

**Case presentation:**

A 20-year old man was presented with back pain, radiculopathy, and urogenital symptoms. Magnetic resonance images revealed an intradural extramedullary mass, isointense in T1-weighted and hyperintense in T2-weighted images, with noticeable post injection enhancement. The clinical and radiological findings simulated neurinoma.

However, a pinkish lesion was removed surgically and histopathological examination revealed lobules of capillary vessels separated by fibrous tissue, suggesting capillary hemangioma.

**Conclusion:**

Although rare and sometimes indistinguishable from more common lesion, capillary hemangioma should be in differential diagnosis of any enhancing intradural extramedullary mass at the level of cauda equina or conus medullaris.

## Background

Tumors of cauda equina account for only 6% of all spinal tumors.[1] The differential diagnosis for enhancing intradural extramedullary lesions in this region includes meningioma, hemangioma, schwannoma, hemangioblastoma, and paraganglioma. [2] Primary hemangiomas of spinal nerve root, mostly affecting the cauda equina, are extremely rare. [3]

In this report, a case of capillary hemangioma of cauda equina is described and different aspects regarding diagnosis, treatment and pathophysiology of the lesion, and differentiation from more common intradural extramedullary conditions are discussed.

## Case presentation

A 20-year old man presented with three months history of low back pain radiating to the legs. He also complained of urinary retention, impotence, and retrograde ejaculation of recent onset. He had bilateral weakness in knee flexion and extension of 3/5. There was also parasthesia of both feet and asymmetrical diminished deep tendon reflexes in both lower extremities, which was worse in right side.

**Figure 1:**
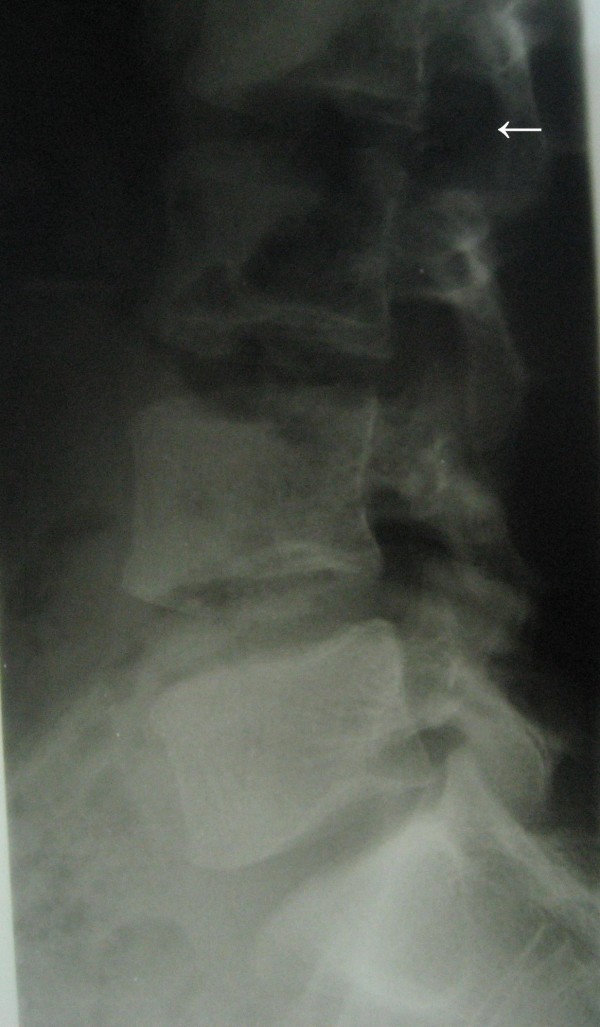
**Plain radiography of lumbar area showing bony erosion and widening of the canal (scalloping) at the level of L3 vertebra (arrow)**.

**Figure 2:**
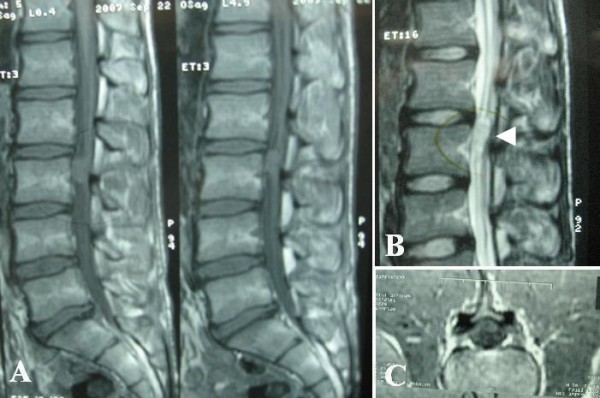
**Sagital magnetic resonance images showing an intradural mass at the level of L3, isointense to spinal cord on T1-weighted (A) and iso-hyperintense on T2-weighted sequences (B)**. T1-weihted axial image demonstrating the intradural mass pushing the caudal roots to the side of the canal **(C)**.

**Figure 3:**
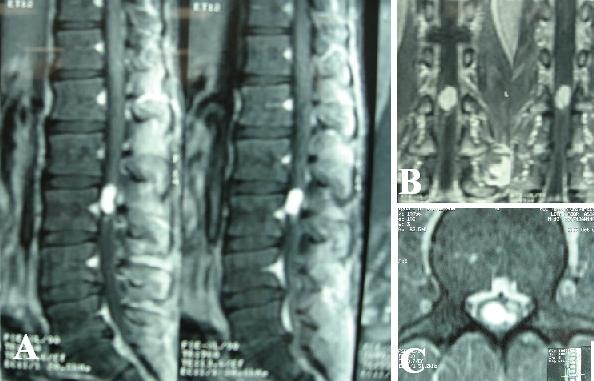
**Post-gadolinium enhancement revealed in sagital (A), coronal (B), and axial (C) magnetic resonance images of this lesion**.

On plain radiography, evidence of vertebral scalloping and widened foramen at the level of L3 was considered probable (Figure 1). Magnetic resonance imaging (MRI) revealed an intradural, well circumscribed mass at the level of L3, isointense to spinal cord on T1-weighted and isointense to hyperintense on T2-weighted sequences (Figure 2). Post gadolinium injection images showed remarkable homogenous enhancement (Figure 3).

Regarding its location and MR appearance, the lesion was considered a cauda equina scwhanoma. Surgical resection was performed through a midline posterior approach. Following a one-level laminectomy at L3, midline duratomy was performed and a pinkish intradural mass was identified. The tumor was attached tightly to one of the rootlets, albeit there was no dural attachment. The adjacent radicular vessels seemed to be enlarged. The mass had squishy consistency with high bleeding tendency. Enbloc resection was performed to reduce blood loss.

**Figure 4:**
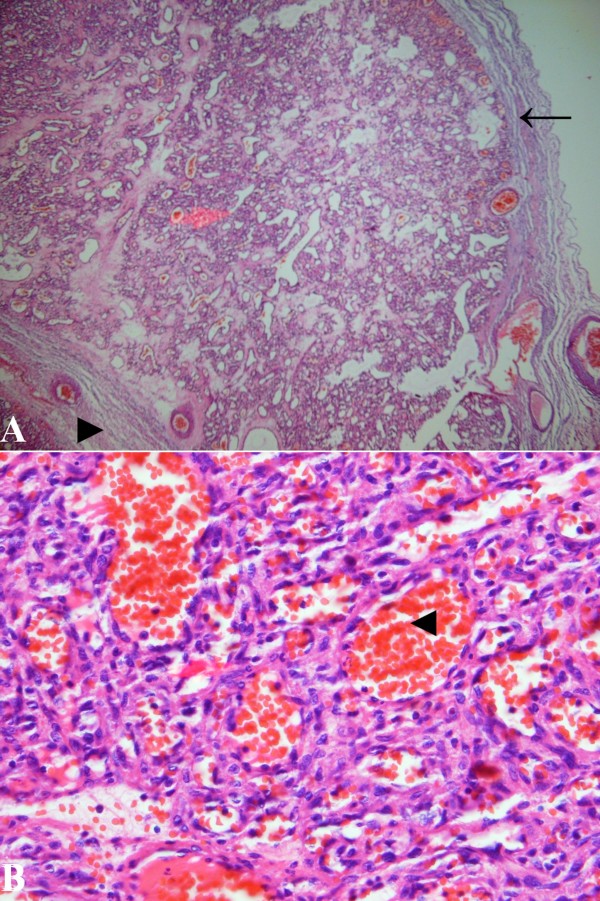
**Photomicrograph of the lesion demonstrating a lobule composing of small capillary vessels lined by a layer of endothelial cells**. The fibrous capsule (arrow), interlobular septa (right-pointing arrowhead), and a capillary lumen (left-pointing arrowhead) are disclosed in the picture. H & E, original magnification, ×4 **(A) **and ×40 **(B)**.

**Figure 5:**
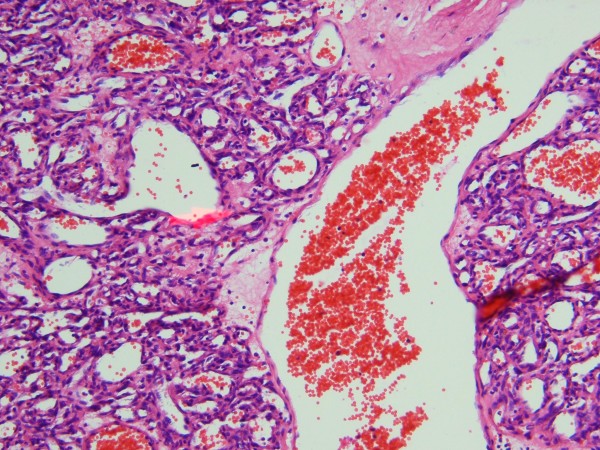
**A large vessel with endothelialized wall in the middle of the specimen, probable to be the feeder vessel. H & E, original magnification, ×20**.

Histopathological assessment revealed encapsulated mass with lobular components containing small size capillary vessels lined by a single layer of flattened endothelial cells (figure 4). There was no evidence of atypia, and the lobules were septated by connective tissue. A large vessel with structured endothelial components was seen in the middle of the sample, most likely to be the feeder vessel (Figure 5).

Following surgery, the patient's motor symptoms were entirely alleviated, and urogenital problems improved marginally. During 1 year follow-up, there was no clinical or radiological evidence of recurrent disease.

## Discussion

Vascular tumors account for about 2% to 7% of all intraspinal tumors. [4] Amongst these lesions, capillary hemangioma in the boundaries of the spinal dura mater is extremely rare, and usually presents distally at the conus medullaris or attached to nerve roots of the cauda equina. [5] Instead, majority of spinal angiomas in these locations are histopatholigically cavernomas. [4] Capillary hemangioma most frequently occurs in cutaneous, subcutaneous, or mucosal tissues as reddish-purple lesions and is more common in children. [6,7] The uncommon intradural condition at the cauda equina occurs in older patients, with equal frequency in men and women. [5]

Haemangiomas of the cauda equina may present with intermittent low back pain, intermittent radicular symptoms, sensory deficits, saddle anaesthesia, paraplegia, sphincter dysfunction, symptoms of hydrocephalus, and subarachnoidal hemorrhage. [4] Indeed, both capillary and cavernous hemangiomas usually behave as space-occupying masses, producing chronic progressive myelopathy or radiculopathy, which may deteriorate acutely following a hemorrhagic episode.[6] This sudden deterioration more often occurs in cavernous angioma, while capillary variant has a slow progressive course.[5] Unusual presentation with increased intracranial pressure, elevated cerebrospinal fluid protein level, and papilledema has been reported in a capillary hemangioma of cauda equina as well.[8]

The diagnosis can be established by MRI. The mass usually appears isointense relative to the spinal cord on T1-weighted, and iso- or hyperintense on T2-weighted MR images. Gadolinium administration results in uniform intense enhancement of the lesion. [3] After a hemorrhagic insult, heterogeneous hyperintensity on T1-weighted images and homogeneous hypointensity on T2-weighted images may correspond with the hemorrhagic focus. [6] The appearance of the enlarged perimedullary draining veins associated with an intraspinal mass on MR images can imply the possibility of an extremely vascular tumor. [2]

The signal characteristics may help differentiate hemangioma from more common intradural extramedullary tumors.[6,7] Meningioma displays isointensity or slight hypointensity on T1-weighted images and isointensity or slight hyperintensity on T2-weighted images, with the mass showing considerable enhancement following gadolinium injection. These signal intensities could assist in differentiation from hemangioma, but the dural tail sign is not useful to distinguish meningioma, since the hemangioma may arise from the inner surface of the dura mater.[6] In intradural extramedullary neurinomas, the signal characteristics are similar to those of intradural hemangioma.[7] Cystic changes or necrosis could be found within neurinomas. In the absence of these features, it is difficult to differentiate neurinoma from hemangioma, as seen in our case. [6] Arteriovenous malformations can be diagnosed through MRI by demonstrating a vascular flow void. [6]

In the spinal intradural extramedullary space, hemangiomas may arise from the blood vessels of the nerve roots in the cauda equina, the inner surface of the dura, or the pial surface of the spinal cord. [6] Two hypotheses have been proposed regarding the pathogenesis of the disease. The lesion may evolve during the early somitic differentiation, at the time of angioblastic differentiation (days 21–24 of embryogenesis), due to impaired movement and differentiation of primitive mesoderm from the embryonic mesodermal plate. [5] Another school of thought claims the origin from vascular structures within the epineurium of the nerve roots affected during individual ontogenesis. [5]

Surgery is the treatment of choice for spinal intradural capillary hemangioma, and most of the symptoms would be reversed postoperatively. [3,5]

On histopathological specimens, capillary hemangiomas usually are of lobulated structures of capillary-sized vessels lined with flattened endothelium, with the lobules being separated by a collagenous stroma. [7] The lesions are surrounded by a thin fibrous pseudocapsule, and there may be a mild lymphocytic infiltrate. [3] Immunohistochemical tests have been positive against neuron-specific enolase and S-100 protein. [3] The tumor cells were shown to stain strongly positively for endothelial markers CD31 and CD34, which are compatible with the diagnosis of capillary hemangioma. [8] These new methods could be of great value in identification of the disease in future.

## Conclusion

Although intradural capillary hemangioma is a rare entity and may be clinically or radiologically indistinguishable from other lesions, it should be considered as the differential diagnosis of any intradural extramedullary mass at the level of conus medullaris or cauda equina.

## Consent

Written informed consent was obtained from the patient and his parents for publication of this case report and accompanying images. A copy of the written consent is available for review by the Editor-in-Chief of this journal.

## Competing interests

The authors declare that they have no competing interests.

## Authors' contributions

SMM made contribution to conception and analyzed the patient data. ZH made contribution in collecting data and drafting the manuscript. MH was contributor in drafting the manuscript. ATM was contributor in revising the manuscript critically.

SAFT has given the final approval for the version to be submitted.
